# Identification and quantitative assessment of motor complications in Parkinson’s disease using the Parkinson’s KinetiGraph™

**DOI:** 10.3389/fnagi.2023.1142268

**Published:** 2023-08-01

**Authors:** Yan Qu, Tingting Zhang, Yunyan Duo, Liling Chen, Xiaohong Li

**Affiliations:** ^1^Department of Neurology, Affiliated Dalian Municipal Friendship Hospital of Dalian Medical University, Dalian, China; ^2^Department of Neurology, First Affiliated Hospital of Dalian Medical University, Dalian, China

**Keywords:** Parkinson’s KinetiGraph™, Parkinson’s disease, motor complications, motor fluctuations, dyskinesia

## Abstract

**Introduction:**

Effective management and therapies for the motor complications of Parkinson’s disease (PD) require appropriate clinical evaluation. The Parkinson’s KinetiGraph™ (PKG) is a wearable biosensor system that can record the motion characteristics of PD objectively and remotely.

**Objective:**

The study aims to investigate the value of PKG in identifying and quantitatively assessing motor complications including motor fluctuations and dyskinesia in the Chinese PD population, as well as the correlation with the clinical scale assessments.

**Methods:**

Eighty-four subjects with PD were recruited and continuously wore the PKG for 7 days. Reports with 7-day output data were provided by the manufacturer, including the fluctuation scores (FS) and dyskinesia scores (DKS). Specialists in movement disorders used the Movement Disorder Society-Unified Parkinson’s Disease Rating Scale-IV (MDS-UPDRS IV), the wearing-off questionnaire 9 (WOQ-9), and the unified dyskinesia rating scale (UDysRS) for the clinical assessment of motor complications. Spearman correlation analyses were used to evaluate the correlation between the FS and DKS recorded by the PKG and the clinical scale assessment results. Receiver operating characteristic (ROC) curves were generated to analyze the sensitivity and specificity of the FS and DKS scores in the identification of PD motor complications.

**Results:**

The FS was significantly positively correlated with the MDS-UPDRS IV motor fluctuation (items 4.3–4.5) scores (*r* = 0.645, *p* < 0.001). ROC curve analysis showed a maximum FS cut-off value of 7.5 to identify motor fluctuation, with a sensitivity of 74.3% and specificity of 87.8%. The DKS was significantly positively correlated with the UDysRS total score (*r* = 0.629, *p* < 0.001) and the UDysRS III score (*r* = 0.634, *p* < 0.001). ROC curve analysis showed that the maximum DKS cut-off value for the diagnosis of dyskinesia was 0.7, with a sensitivity of 83.3% and a specificity of 83.3%.

**Conclusion:**

The PKG assessment of motor complications in the PD population analyzed in this study has a significant correlation with the clinical scale assessment, high sensitivity, and high specificity. Compared with clinical evaluations, PKG can objectively, quantitatively, and remotely identify and assess motor complications in PD, providing a good objective recording for managing motor complications.

## Introduction

Parkinson’s disease (PD) is a common neurodegenerative disorder that seriously affects the quality of life and social function. With disease progression and dopamine drug application, people with PD (PwPD) may develop motor complications such as motor fluctuations and dyskinesia (Ahlskog and Muenter, 2001; Stocchi et al., 2010). The incidence of motor complications is approximately 3% after 1 year of treatment, 41% after 6 years, and 70% after 9 years (Ahlskog and Muenter, 2001). It was reported that after 5 to 10 years of levodopa treatment, approximately 57 to 90% of PwPD experience motor complications (Kum et al., 2009). The disability caused by motor fluctuations and dyskinesia is sometimes more severe than the motor impairment of PD itself. Early identification and assessment of motor complications are crucial guidelines for optimizing treatment and improving motor function.

Currently, the available assessment of motor complications in PD depends on clinical assessment scales such as the Movement Disorder Society-Unified Parkinson’s Disease Rating Scale part IV (MDS-UPDRS IV) (Goetz et al., 2008b), the wearing-off questionnaire 9 (WOQ-9) (Stacy et al., 2008), the unified dyskinesia rating scale (UDysRS) (Goetz et al., 2008a). These results are obtained through face-to-face observations, and their accuracy is limited by the subjectivity of the assessing physician. Moreover, motor symptom diaries completed by PwPD are also widely used in clinical practice to help document motor fluctuations and dyskinesia (Hauser et al., 2000). Diaries require PwPD to understand the meaning of motor fluctuations, to distinguish between dyskinesia and tremor, and require training before recording. The extremely heavy workload tends to mean fatigue and reduced compliance, while recalling bias and self-reported subjectivity further reduce diaries’ accuracy (Hauser et al., 2004).

**FIGURE 1:**
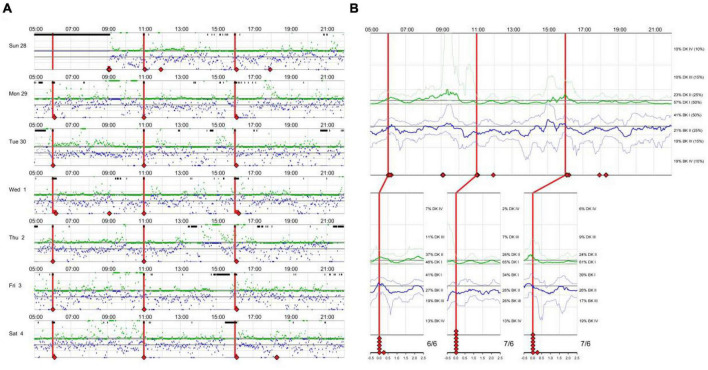
An example of Parkinson’s KinetiGraph daily plot **(A)** and summary plot **(B)** in a patient.

There is an eager necessity to develop accurate, objective, and continuously recordable tools to evaluate motor complications in PD. Nowadays, these clinical challenges can be addressed by applying biosensing systems and wearable ambulatory continuous objective monitoring (COM) technologies. The Parkinson’s KinetiGraph™ (PKG™, Global Kinetics Corporation, Australia) (Griffiths et al., 2012), is one of the representative products, which is the first wearable device approved by the Food and Drug Administration (FDA) for clinical evaluation in PD. The PKG strap is ergonomically designed, adjustable in tightness, and can provide comfortable wear for an extended period. This device can provide a continuous, visual, and remote record including scaled measurement of bradykinesia, motor fluctuations, and dyskinesia and correlation with medication (Hauser et al., 2004; Griffiths et al., 2012; Farzanehfar and Horne, 2017). An example is shown in Figure 1.

Several studies have shown promising results in using the PKG to assess motor symptoms in PwPD. The PKG reliably and objectively evaluates motor symptoms, including bradykinesia, tremor, fluctuations, and dyskinesia, especially subtle changes in movement that may go unnoticed during clinical visits (Griffiths et al., 2012; Horne et al., 2015; Farzanehfar and Horne, 2017; Pahwa et al., 2020; Isaacson et al., 2022). These studies also highlight the value of PKG data in understanding disease progression and treatment response (Guan et al., 2021; Sundgren et al., 2021; Watts et al., 2021). Overall, PKG demonstrates great potential as a valuable tool for personalized precision medicine and improving prognosis in PwPD.

Regrettably, few studies have focused on the role of PKG in the assessment of dyskinesia, especially in the Chinese PD population. The incidence of dyskinesia in PD varies significantly among the countries reported. The prevalence of dyskinesia in Parkinson’s disease within 10 years of onset was 59% in Europe and the United States (Sharma et al., 2010), whereas the prevalence of dyskinesia was found to be about 8.6% in a Chinese study (Zhang et al., 2014). The research gap of PKG in the Chinese PD population remains to be bridged.

While significant advances have been made in wearable ambulatory COM technologies, many clinicians remain uncertain of how to incorporate them into clinical practice, including the value to clinical decision-making. Our study, the first study to simultaneously investigate the value of PKG in identifying motor fluctuations and dyskinesia in the Chinese PD population, aims to fill the data gap in this area, and hopefully to further incorporate wearable biosensing devices into clinical practice.

## Materials and methods

### Participants

A total of 84 subjects who attended the PD specialist outpatient clinic in the Department of Neurology, Affiliated Dalian Municipal Friendship Hospital of Dalian Medical University, from 2019 to 2020 were recruited. All subjects signed an informed consent form. This study was approved by the Ethics Committee of Affiliated Dalian Municipal Friendship Hospital of Dalian Medical University (Ethical Approval Number: YY-LL-2022-015). The inclusion criteria were: meeting the MDS diagnostic criteria for clinically established PD (Postuma et al., 2015) and Hoehn and Yahr (H-Y) stage 1–4. The exclusion criteria were: (1) parkinsonism-plus syndrome or secondary parkinsonism; (2) history of severe brain atrophy, post-traumatic brain injury, hydrocephalus, cerebrovascular disease, etc.; (3) severe cognitive impairment with mini-mental state examination (MMSE) ≤ 20 scores; (4) non-PD physical disability; (5) inability to wear the PKG (lack of compliance or other conditions that make the PwPD unable to wear the device) or inability to complete the clinician assessment. Based on the results of the WOQ-9 scale, we divided the subjects into PD fluctuators (score ≥ 1) and non-fluctuators (score = 0). Meanwhile, we grouped the subjects into PD with dyskinesia (score ≥ 1) and non-dyskinesia (score = 0) on the basis of the MDS-UPDRS IV (4.1 + 4.2) scores.

### Methods

A physician specializing in movement disorders was responsible for distributing the PKG and coping with the data analysis and interpretation. One charge can support continuous data collection for 7 days of continuous wear. The PKG should fit snugly on the wrist to ensure accurate and reliable data collection, while maintaining an appropriate level of looseness. In clinic visits, the subjects were given the PKG with detailed instructions and wore it for 7 consecutive days. At the end of wearing, two movement disorder specialists independently assessed PwPD by using the MDS-UPDRS IV, the WOQ-9, and the UDysRS. The scales were evaluated during the medication “ON” stage for the PwPD’s better status and compliance. These scales reflect the presence or absence of motor fluctuations and dyskinesia over the past week, and the scales immediately following the 7-day PKG wearing can be considered the same time period were evaluated. The mean scores were included in the statistical analysis as the results of the scale scores.

### Data collection

Data collection included sex, age, disease duration, Hoehn and Yahr (H-Y) stage, and clinical scale results. The scale scores are as follows.

(1) Motor fluctuations: The neurologist assessments and the WOQ-9 scale were used to determine the presence or absence of motor fluctuations. The WOQ-9 was recommended by MDS to assess the presence of motor fluctuations: a “yes” in columns 1 and 2 of questions 1, 2, 4, 6, and 9 indicates the presence of motor fluctuations (Stacy et al., 2008). The MDS-UPDRS IV motor fluctuation score (items 4.3–4.5) was used to assess the severity of motor fluctuations. Those three 5-point items cover wearing-off duration, functional impact, and complexity, with higher scores indicating greater severity (Goetz et al., 2008b; Antonini et al., 2011).
(2) Dyskinesia: The MDS-UPDRS IV dyskinesia score (items 4.1 and 4.2) was used to assess the presence of dyskinesia, with a score of ≥ 1 indicating the presence of dyskinesia (Goetz et al., 2008b). The UDysRS total score and the UDysRS III score (Part III score: objective evaluation of the severity of impairment due to dyskinesia): the higher the score, the greater the severity (Goetz et al., 2008a).

### PKG data acquisition and analysis

The PKG (GKC-2000, Global Kinetics, Melbourne, Australia) (Griffiths et al., 2012) proprietary system consists of a watch and a data analysis platform. The PKG uses a precise digital accelerometer and gyroscope to collect motion data. The PwPD wears the device on the side with the heavier tremor (or the right hand if there is no tremor) for 7 days (7 h × 24 h). The PKG automatically collects motion data from the patient every 2 min. After wearing the PKG, the data are transmitted to the data analysis platform. Then the basic motion data are analyzed using a patented mathematical algorithm to produce a report with parameters such as the bradykinesia score (BKS), the dyskinesia score (DKS), the fluctuation score (FS), the percent time tremor (PTT), and the percent time immobile (PTI) (Griffiths et al., 2012).

Bradykinesia score (BKS): The PKG calculates the BKS based on the maximum acceleration recorded every 2 min of motion and the mean spectral power (MSP) of that peak acceleration (Rovini et al., 2019). The rationale is that people without bradykinesia typically have higher accelerations and energy during movement compared to those with bradykinesia (Griffiths et al., 2012).

Dyskinesia score (DKS): A calculation of DKS based on the mean acceleration and the MSP generated in a 2 min epoch. The rationale was that individuals with dyskinesia would have a higher MSP in slower movements compared to normal subjects, and they would also spend more time making movements with acceleration greater than the mean than normal subjects (Griffiths et al., 2012).

There were 270 BKS and DKS generated from 09:00 to 18:00 each day, for a total of 1,890 over 7 days. The median BKS and DKS were used as an indicator of the severity of bradykinesia and dyskinesia, respectively.

Fluctuation score (FS): The FS is a logarithm of the sum of the interquartile distances of all recorded BKS and DKS, which can reflect the dispersion of variables stably and is suitable for motor fluctuation. This value had been validated in the PD cohort (Horne et al., 2015).

### Statistical analysis

We used SPSS Statistics 27.0 (IBM Corp., Armonk, NY, USA) for statistical analysis. Data conforming to normal distribution are presented as mean ± standard deviation (X ± S), and those with a non-normal distribution are expressed as median and interquartile range. We used Pearson correlation analyses for normally distributed data and Spearman correlation analyses for non-normally distributed data. We employed independent samples *t*-tests and Mann–Whitney U tests to compare the differences between groups for normally and non-normally distributed data, respectively. For all analyses, we considered *p* < 0.05 to be a statistically significant difference. We generated receiver operating characteristic (ROC) curves and determined the area under the curve, the Youden index, the best bounds, the sensitivity, and the specificity of the FS and DKS.

## Results

### Study population

We enrolled 84 participants in the study at the time of this analysis. Demographics information, clinical scale measurements, and PKG parameters of these subjects are shown in Table 1. A total of 84 subjects were assessed using the WOQ-9, and 35 subjects with a score of ≥ 1 were considered PD fluctuators, 49 subjects with a score of 0 as non-fluctuators. Using the MDS-UPDRS IV (4.1 + 4.2) assessment, 30 subjects with a score of ≥ 1 were considered as PD with dyskinesia, and 54 subjects with a score of 0 as non-dyskinesia. The results were further determined with neurologists’ assessments. The number of PD with motor fluctuations is 35, with dyskinesia is 30, and with both is 17.

**TABLE 1:** Demographics information, clinical scale measurements, and PKG parameters of participants.

Item	PD
Numbers	84
Sex (male/female)	51/33
Age (year)	69.89 ± 9.90
Disease duration (year)	5 (3, 7)
H-Y stage	3 (2.5, 3)
**With motor complications [*n* (%)]**	
Motor fluctuations^a^	35 (41.67)
Dyskinesia^b^	30 (35.71)
MDS-UPDRS IV (4.3 + 4.4 + 4.5)	0 (0, 4.00)
UDysRS total	0 (0, 27.50)
UDysRS III	0 (0, 7.00)
**PKG parameters**	
BKS	33.96 ± 6.63
FS	6.65 (5.43, 8.53)
DKS	0.50 (0.20, 1.10)

^a^A total of 84 subjects were assessed using the WOQ-9, and 35 subjects with a score of ≥ 1 were considered as PD with motor fluctuations.

^b^A total of 84 subjects were assessed using the MDS-UPDRS IV (4.1 + 4.2), and 30 subjects with a score of ≥ 1 were considered as PD with dyskinesia.

H-Y stage, Hoehn and Yahr stage; WOQ-9, wearing-off questionnaire 9; MDS-UPDRS IV, the part IV of the Movement Disorder Society-Unified Parkinson’s Disease Rating Scale; UDysRS total, the total of the unified dyskinesia rating scale; UDysRS III, the part III of the unified dyskinesia rating scale; BKS, bradykinesia score; FS, fluctuation score; DKS, dyskinesia score.

### Correlation of FS with MDS-UPDRS IV motor fluctuation scales

**FIGURE 2:**
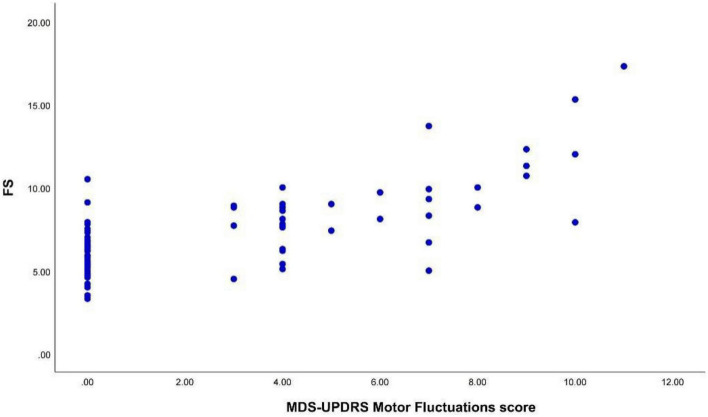
The FS was positively correlated with the MDS-UPDRS IV motor fluctuation scores (*r* = 0.645, *p* < 0.001). FS, fluctuation score; MDS-UPDRS IV, the part IV of the Movement Disorder Society-Unified Parkinson’s Disease Rating Scale.

Spearman correlation analysis revealed a positive and significant correlation between the FS and the MDS-UPDRS IV motor fluctuation (items 4.3–4.5) scores (*r* = 0.645, *p* < 0.001) (Figure 2).

### FS for diagnosis and assessment of motor fluctuations

We used the WOQ-9 scale and neurologist assessments to divide the subjects into two groups. We compared the intergroup variability of the FS between the PD fluctuators and non-fluctuators. The Mann–Whitney U test revealed that the difference between the two groups was statistically significant (*p* < 0.001), and higher FS in the PD fluctuators than in the PD non-fluctuators (Table 2).

**TABLE 2:** Comparison of FS between the PD fluctuators and non-fluctuators group.

Group	*n*	FS
Fluctuators	35	8.60 (7.40, 10.00)
Non-fluctuators	49	6.20 (5.25, 6.80)
*z* value	−4.856	
*p*-value	< 0.001	

Using the Mann–Whitney test, *p* < 0.05 is considered a statistically significant difference. FS: fluctuation score.

**FIGURE 3:**
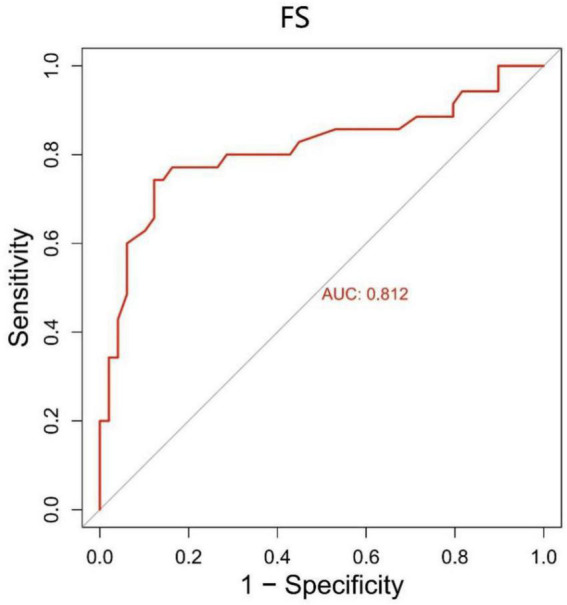
The ROC curve for the FS with a sensitivity of 74.3% and a specificity of 87.8%. The area under the curve (AUC) was 0.812 (*p* < 0.001). FS, fluctuation score.

We constructed a ROC curve for the FS with the sensitivity and 1- specificity (Figure 3). The area under the curve (AUC) was 0.812 (*p* < 0.001). This resulted in a maximum Youden index of 62.04%, which corresponds to an optimal cut-off value of 7.5 for the FS. Patients with a higher score are suggested to have motor fluctuations. The analysis yielded a sensitivity of 74.3% and a specificity of 87.8%.

### Correlation of DKS with UDysRS total and UDysRS III scales

**FIGURE 4:**
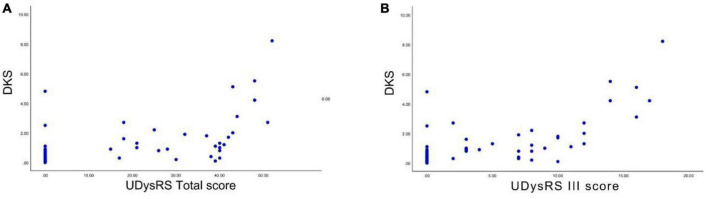
Correlation between DKS and UDysRS total score and UDysRS III score. **(A)** The DKS was positively correlated with the UDysRS total score. **(B)** The DKS was positively correlated with the UDysRS III score (*r* = 0.634, *p* < 0.001). DKS, dyskinesia score; UDysRS III, the part III of the unified dyskinesia rating scale.

We performed Spearman correlation analyses between the DKS and the UDysRS total score and between the DKS and the UDysRS III score. There was a positive and significant correlation between the DKS and the UDysRS total score (*r* = 0.629, *p* < 0.001) (Figure 4A). There was also a positive and significant correlation between the DKS and the UDysRS III score (*r* = 0.634, *p* < 0.001) (Figure 4B).

### DKS for diagnosis and assessment of dyskinesia

Movement Disorder Society-Unified Parkinson’s Disease Rating Scale-IV (MDS-UPDRS IV) recommended by MDS for the assessment of dyskinesia, We divided the 84 PwPD into the PD dyskinesia and non-dyskinesia group. We compared the intergroup variability of the DKS between the PD dyskinesia and non-dyskinesia groups. Based on the Mann–Whitney U test, the difference was significant. The DKS in the PD dyskinesia group were significantly higher than those in the PD non-dyskinesia group (*p* < 0.001) (Table 3).

**TABLE 3:** Comparison of DKS between the PD with dyskinesia and non-dyskinesia group.

Group	*n*	DKS
Dyskinesia	30	1.30 (0.80, 2.70)
Non-dyskinesia	54	0.30 (0.10, 0.60)
*z* value	−5.488	
*p*-value	< 0.001	

Using the Mann–Whitney test, *p* < 0.05 is considered a statistically significant difference. DKS, dyskinesia score.

**FIGURE 5:**
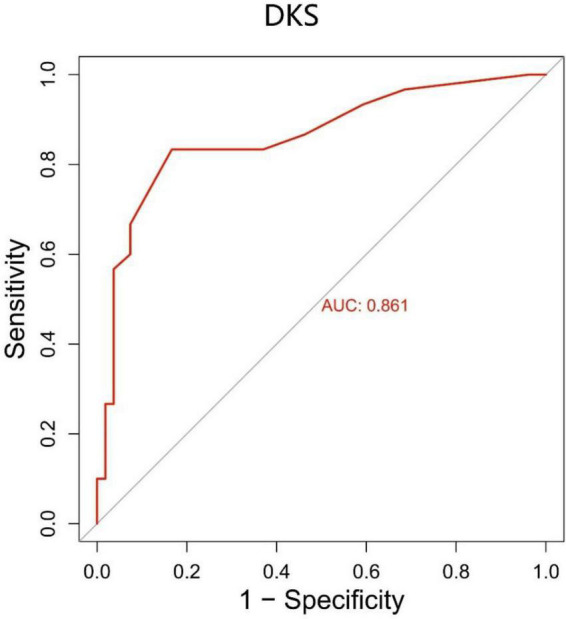
The ROC curve for the DKS with a sensitivity of 83.3% and a specificity of 83.3%. The area under the curve (AUC) was 0.861 (*p* < 0.001). DKS, dyskinesia score.

We generated a ROC curve for the DKS with sensitivity and 1-specificity (Figure 5). The AUC was 0.861 (*p* < 0.001). This resulted in a maximum Youden index of 66.67%, which corresponds to an optimal cut-off value of 0.7 for the DKS. PwPD with a higher score are suggested to have dyskinesia. The DKS had a sensitivity of 83.3% and a specificity of 83.3%.

## Discussion

We compared the data obtained from the PKG with specialists’ assessment of motor complications in PD. Correlation analysis indicated a significant positive correlation between the FS and the MDS-UPDRS IV motor fluctuation scores and a significant positive correlation between the DKS and the UDysRS total score and the UDysRS III score. The ROC curves suggest that the FS and DKS have significantly high sensitivity and specificity. These results indicate that the PKG can be applied for objective, dynamic, remote and quantitative assessment of motor fluctuations and dyskinesia in PD.

Motor fluctuations, particularly the wearing-off phenomenon, are common complications in PD characterized by a reduction in medication duration. The prevalence of motor fluctuations within 1 year of PD onset was reported to be 29.0% in a 2014 multicenter study, while it reached as high as 60.3% in those with a disease duration of ≥ 5 years (Chen et al., 2014). Dyskinesia, characterized by involuntary movements in response to dopaminergic stimulation, includes hyperactivity, head shaking, facial and oral movements, and dance-like movements of the limbs, neck, and trunk. The prevalence of dyskinesia in Chinese PD patients using levodopa was found to be 57–90% in a cross-sectional study in Hong Kong (Kum et al., 2009), with the prevalence increasing over time (Tran et al., 2018). However, compared to symptoms like bradykinesia and tremor, detecting fluctuations and dyskinesia, especially when symptoms are less obvious and can be confused with tremor, is challenging for both clinicians and patients.

The PKG (Griffiths et al., 2012) uses portable sensing technology to collect real-time acceleration measurements of the limbs of patients with PD and analyzes them using a validated algorithm. A single wearing allows PwPD to be recorded for motor symptoms for 7 consecutive days, overcoming the limitations of subjective and non-continuous assessment of the scale. PD motor parameters obtained by applying the PKG include BKS, PTT, DKS, FS, and PTI. We previously evaluated two of these parameters, the BKS and PTT, for quantitative assessment of motor bradykinesia and tremor symptoms in PD (Qu et al., 2023). In this study, we focused on the FS and DKS provided by the PKG.

Fluctuation scores (FS) was significantly and positively correlated with MDS-UPDRS IV motor fluctuations (items 4.3–4.5) scores, suggesting that FS was consistent with the scale measures. The more severe motor fluctuations were associated with higher FS.

We used WOQ-9 scores and neurologist assessments to separate patients. Based on ROC curve analysis, we found that FS had high sensitivity and specificity (74.3% sensitivity and 87.8% specificity) with an optimal cutoff value of 7.5; higher values indicated motor fluctuations in PD patients. Our finding is consistent with another study (Horne et al., 2015). Those authors identified fluctuators and non-fluctuators through clinical interviews used to test the ability of FS values to distinguish between the two groups. The results they obtained were significant, with a critical FS value of 7.7. This is generally consistent with our findings, suggesting that FS obtained by BKS and DKS has the potential as a tool for identifying motor fluctuations and optimizing the treatment of PD.

The WOQ-9 scale identified fluctuations in Parkinson’s disease with a sensitivity of 96% but a specificity of only 41% (Stacy et al., 2008). Due to the lack of a simpler and more specific assessment method, the WOQ-9 is still used as the “Recommended” assessment scale for diagnostic screening for wearing-off by the Movement Disorder Society in 2011 despite its poor specificity (Antonini et al., 2011). Our study of the PKG yielded a specificity of 87.8%, which is significantly higher than the specificity of the WOQ-9, offering significant advantages.

In this study, we evaluated the severity of dyskinesia by using the UDysRS total score and the UDysRS III score; both were positively correlated with the DKS. This suggests that the DKS is consistent with the scale measures. The development and validation of the PKG (Griffiths et al., 2012) involved correlating the DKS with the abnormal involuntary movement scale (AIMS) scores. Horne et al. (2016) validated the DKS parameters by correlating the DKS values with the AIMS score in 40 subjects and concluded that the DKS is positively correlated with the AIMS scores. We have validated the consistency of the PKG parameters by assessing dyskinesia scales other than the AIMS, further supporting the utilization of the PKG as a consistent, valid, and reliable measure to assess dyskinesia.

The PKG has other significant advantages over clinical scales in the assessment of dyskinesia. An expert panel formed to evaluate the role of the PKG in the routine clinical assessment of PD concluded that because the PKG can provide important information about the relationship between dyskinesia and drug intake, it can help distinguish the types of dyskinesia (Pahwa et al., 2018). It is possible to distinguish between peak and biphasic dyskinesia as well as between ON-stage and OFF-stage dyskinesia. In addition, “tremor” and “dyskinesia” can be confused clinically, and (Braybrook et al., 2016) reported that PKG-supported spectral analysis can distinguish between tremor and dyskinesia. Tremor spectral analysis shows a distinct primary peak > 3 Hz, whereas dyskinesia spectral analysis displays energy in a wide frequency range of 0.1–8 Hz, often without a distinct peak. This is highly meaningful for better clinical practice.

In this study, we applied the MDS-UPDRS IV (items 4.1 and 4.2) score as a criterion and based on ROC curve analysis, the DKS has an optimal cut-off value of 0.7, a sensitivity of 83.3%, and a specificity of 83.3%. This threshold differs significantly from the DKS cut-off values reported in previous studies outside of China. Currently, several studies (Farzanehfar et al., 2018; Odin et al., 2018; Pahwa et al., 2018) have used a DKS of 9 as the cut-off value, and DKS ≥ 9 is considered to indicate the presence of dyskinesia. They considered dyskinesia to be “manageable” if DKS < 9, which was associated with an AIMS scale < 10. Nevertheless, in their large multi-country-centered PKG database, (Pahwa et al., 2020) found some differences in the median DKS of patients with PD and the proportion of patients with DKS > 9 in different countries (Table 4). They analyzed the differences between countries maybe in relation to different patterns of medication interventions, physician treatment levels, and national insurance coverage policies.

**TABLE 4:** PKG parameters of PD in various countries.

	Median DKS	DKS > 9 percentage	Median BKS
Australia (*n* = 8,506)	1.8	9.5%	25.8
United Kingdom (*n* = 5,614)	1.8	11.5%	26.5
United States (*n* = 4,729)	1.2	5.4%	27.5
Sweden (*n* = 2,782)	2.3	14.7%	25.0
Germany (*n* = 2,070)	1.6	8.5%	26.7
Netherlands (*n* = 1,641)	1.9	9.6%	26.0
France (*n* = 770)	2.8	14.8%	24.6
**Our center in China (*n* = 84)**	**0.5**	**0**	**33.96**

DKS, dyskinesia score; BKS, bradykinesia score.

Our analysis of 84 subjects with PD yielded a cut-off value of 0.7 and a median of 0.5 for the DKS, which are significantly lower than in studies from other countries. A higher BKS of 33.96 than in other countries of 24.6∼27.5. It is worth mentioning that the prevalence of dyskinesia varies significantly in different countries. The prevalence of dyskinesia in Parkinson’s disease patients within 10 years of onset was 59% in Europe and the United States (Sharma et al., 2010), whereas the prevalence of dyskinesia was found to be about 8.6% in Chinese PD (Zhang et al., 2014). This difference may stem from the much higher doses of dopaminergic medications taken by Western patients over the same period than in China. We propose several possible reasons as follows. First, there is a notable difference in the sample size. We were a single-center study and considered only 84 cases (a small number due to inconvenient access to care due to the COVID-19 pandemic), compared with 770–8,506 cases in databases from other countries. This may explain the significant difference in the median and critical values of DKS. There is a necessity to pool multicenter studies from China to clarify the distribution of DKS in the Chinese PD population. Second, the scale used in our study differs from the AIMS scale used in other studies, which may lead to some differences in the results. Given the disparities among different countries and races, further research is necessary to investigate if height and arm length have potential impacts on arm acceleration during the act of walking. On the other hand, we consider the high median BKS and the low median DKS in this study to be associated with greater fear of adverse drug reactions in Chinese PwPD and the cautious treatment decisions of the physicians in our center. This may lead to an inadequate improvement in bradykinesia (higher BKS scores) but a lower incidence and degree of dyskinesia in patients with PD at our center.

There are limitations to this study. The single-center, small-sample study makes it necessary to be more cautious in the interpretation of our conclusions. The duration of disease, age at onset, and duration of medication may influence the incidence of motor complications, which were not further stratified in our study. Clinical assessments were based on the scales conducted in the ON stage after medication with a better status, which may have influenced our study to some extent. This is regrettable that we did not distinguish between the ON and OFF stage. There are some methodological differences between our study and other studies, which makes it important to be cautious when comparing our findings with those of other studies. In addition, the PKG is limited in assessing motor symptoms because it is worn only in the more severely affected arm, reducing its ability to measure bilateral or axial symptoms and thus making the overall measurement of motor symptom progression challenging.

## Conclusion

In summary, in the PD population analyzed in this study, the PKG has a good correlation with clinical scales and high sensitivity and specificity for the identification and diagnosis of motor fluctuations and dyskinesia. The FS can effectively assess motor fluctuation symptoms and the DKS may effectively assess the dyskinesia symptoms in PD. Compared with the clinical scale assessment, the application of the PKG to monitor the motor complications of PD has the advantages of objective, quantitative, and dynamic assessment, which can meet the urgent need for remote recording and management of these people. Therefore, we consider that application in the clinical practice of the PKG can provide clinically meaningful data to aid clinical decision-making, thereby benefiting the PD population.

## Data availability statement

The original contributions presented in this study are included in the article/supplementary material, further inquiries can be directed to the corresponding author.

## Ethics statement

The studies involving human participants were reviewed and approved by the Ethics Committee of Affiliated Dalian Municipal Friendship Hospital of Dalian Medical University. The patients/participants provided their written informed consent to participate in this study.

## Author contributions

YQ, TZ, and XL designed the study and wrote and polished the manuscript. YQ, YD, and LC performed this research and the statistical analysis. All authors reviewed the manuscript and gave the final approval for publication.
